# Multi-Arm Global Cooperative Coal Gangue Sorting Method Based on Improved Hungarian Algorithm

**DOI:** 10.3390/s22207987

**Published:** 2022-10-19

**Authors:** Hongwei Ma, Xiaorong Wei, Peng Wang, Ye Zhang, Xiangang Cao, Wenjian Zhou

**Affiliations:** 1 School of Mechanical Engineering, Xi’an University of Science and Technology, Xi’an 710054, China; 2 Shaanxi Key Laboratory of Mine Electromechanical Equipment Intelligent Detection and Control, Xi’an 710054, China

**Keywords:** coal gangue sorting, global work, multi-arm collaboration, task assignment, Hungarian algorithm

## Abstract

The existing multi-manipulator sorting method for gangue that utilizes a multi-task allocation strategy is not satisfactory. The single manipulator working space is fixed, lowering the cooperation degree between the manipulators and leading to a low sorting rate. Therefore, this paper proposes a multi-manipulator cooperative sorting method that can work globally. First, a benefit function based on the sorting time and quality of the gangue is constructed by combining the gangue flow information and the manipulator state. The time parameter is obtained via the manipulator’s dynamic target tracking trajectory planning algorithm based on PID control. Secondly, the benefits matrix is standardized and updated many times to improve the Hungarian algorithm to achieve task allocation, and the initial solution with priority is obtained. Finally, the solutions are analyzed and processed cooperatively in order of priority. The conflicts between multiple robotic arms are eliminated through task cooperation and trajectory cooperation until the sorting task that the robot arm can execute is obtained from the allocation results. Experiments involving different sorting methods were completed on a multi-arm coal and gangue sorting experimental robot platform. The experimental results show that the sorting efficiency of the proposed method is about 10% and 20% higher than that of the fixed space dynamic and designated space fixed points methods, respectively, under different belt speeds. This method can guarantee system benefits, effectively implements cooperative control of multi-manipulator operations in the whole area, and improves the efficiency of coal gangue sorting.

## 1. Introduction

Coal gangue accounts for about 15% of coal content, and burning it produces harmful substances and affects the efficiency of coal combustion. The existence of coal gangue seriously restricts coal selection rates and quality, so the separation of coal gangue is a vital step in coal production and is an important method of improving coal quality [1,2]. The existing coal gangue sorting methods have problems such as high labor intensity of workers, serious waste of water resources, and radiation of rays. With the development of machine vision and robot technology, image processing technology, and intelligent control technology, the use of robots to sort coal gangue has become a new research direction [3,4,5]. The process of sorting coal gangue with robots mainly involves two parts: recognition and tracking/grasping. To date, there have been many studies on the recognition of gangue using image processing and pattern recognition, and good results have been achieved [6,7,8,9]. The research on tracking and grasping focuses on the trajectory planning problem of a single manipulator end-effector tracking a dynamic target [10,11,12]. However, the coal gangue has a large volume, fast movement speed, and many sorting targets. It is difficult for a single manipulator to complete such a complex task, and the sorting rate is low. The collaborative operation of multiple manipulators has better environmental adaptability and fault tolerance. It can achieve efficient task matching and joint control, so multi-arm cooperative sorting has gradually become a research hotspot [13,14].

Multi-arm cooperative sorting is closely related to task allocation and trajectory planning, and task allocation is the primary problem for achieving multi-arm cooperation [15,16]. According to the relevant literature in recent years, task allocation strategies are mostly used in UAV formation, intelligent warehousing, resource scheduling, firefighting, and other environments [17,18,19,20]. There are relatively few studies on multi-dynamic objective task allocation for coal gangue sorting. Wang et al. [21] designed a multi-arm coal gangue sorting robot system based on machine vision, laying a theoretical foundation for the robot sorting of coal gangue. Cao et al. [22] proposed a method for the cooperative sorting of coal gangue by multiple robotic arms and realized the autonomous planning of the trajectory of the robotic arms to pick up the gangue. Wu et al. [23] proposed a multi-arm collaborative strategy to solve the multi-objective assignment problem of gangue flow. Zhao et al. [11] designed a coal gangue sorting robot with two parallel arms, which significantly shortened the sorting cycle. Sun et al. [24] designed a multi-objective motion planning model for a robot, which enables the robot to remove as much gangue as possible in a limited time and improves the accuracy and efficiency of coal gangue sorting. However, the existing research on coal gangue sorting has the following shortcomings: (1) The principle of first come first sort is adopted in the task allocation strategy, and the benefit function between the gangue flow and manipulator is not established for task allocation from the perspective of system income, so the maximum system benefit cannot be guaranteed. (2) Each manipulator has a fixed working space, and the manipulator only needs to sort the gangue in its own working space. The constraint conditions of the manipulator are relatively few in number, and the cooperation degree between the manipulators is low. (3) The influence of the placement point’s choice on the task’s completion is not considered when each manipulator works continuously.

Aiming at the above problems, this paper proposes a method for the collaborative sorting of coal gangue under the global working mode of multiple manipulators. This method can effectively complete task allocation, realize the continuous sorting of multiple manipulators over the whole working space, ensure the efficient cooperation of multiple manipulators, and improve the sorting efficiency of coal gangue.

The main contributions of this paper are as follows:

1. A cooperative coal gangue sorting method is proposed under a global multi-arm working mode. This method allows multiple robot arms to share the same sorting space. The cooperation degree of multiple robot arms is higher.
2. The benefit function is established from the perspective of system benefits, and the Hungarian algorithm is improved by standardizing and repeatedly updating the benefits matrix to carry out multi-objective task allocation. Real-time allocation results with priorities are obtained.
3. In this paper, the concept of task collaboration and trajectory collaboration is proposed. In order of priority, collaborative processing is carried out until the optimal feasible tasks are determined to reduce the computational burden and to ensure the system’s revenue.

The rest of this paper is organized as follows. In Section 2, we introduce the working principles of a multi-arm gangue sorting robot. In Section 3, we introduce the real-time task allocation method based on comprehensive income and its solving process. In Section 4, we introduce the cooperative process of the multi-manipulator and the evaluation index of the sorting result. In Section 5, we conduct related experiments and analyze the results. The thesis is summarized in Section 6.

## 2. Working Principle of Multi-Arm Coal and Gangue Sorting Robot

### 2.1. Introduction to the Multi-Arm Coal and Gangue Sorting Robot

**Figure 1:**
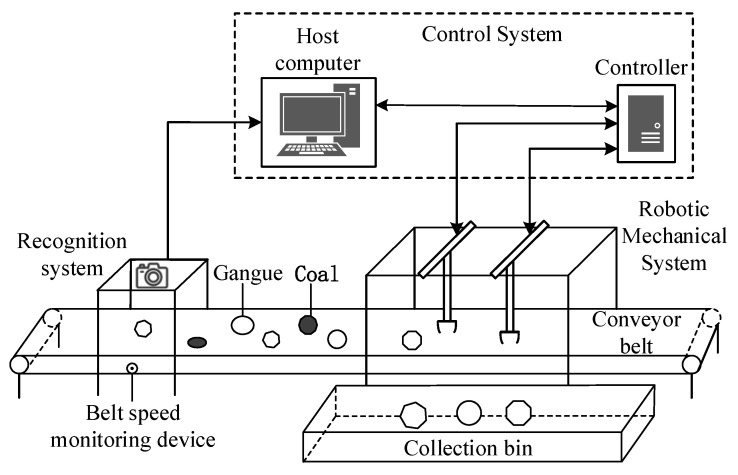
Multi-arm coal and gangue sorting robot system.

Given the characteristics of gangue, which include a large mass and fast speed, a truss-type multi-arm coal and gangue sorting robot is selected in this paper. The multiple arms are placed above the conveyor belt and share the same guide rail, and the manipulator arms are isomorphic. The multi-arm coal and gangue sorting robot is composed of a recognition system, control system, mechanical robot system, etc., as shown in Figure 1.

The recognition system obtains the position coordinates, quality parameters, and other information about the gangue and sends it to the host computer. The upper computer calculates the coordinate information in real time according to the speed of the belt conveyor to screen out the gangue to be sorted. Based on the collaborative sorting strategy, the multi-arm system implements multi-objective task allocation and multi-arm cooperative processing and sends the feasible results calculated in real time to the controller to guide the corresponding manipulator’s work.

### 2.2. Cooperative Sorting Principle of Coal Gangue Sorting Robot with Multiple Manipulators

**Figure 2:**
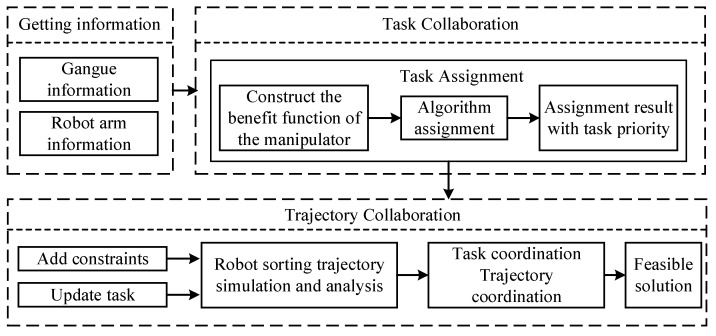
Cooperative sorting principles of multi-arm coal and gangue sorting robot.

In this paper, the conflict types in the sorting process are divided into task conflict and trajectory conflict. The collaborative sorting process is divided into task cooperation and trajectory cooperation. A task conflict is defined as a conflict that cannot be eliminated by trajectory planning, in which case, the task needs to be replaced. A trajectory conflict is a conflict between robot arms when completing their sorting tasks through trajectory planning. Task coordination refers to changing the assignment result of task allocation to determine a feasible solution. Trajectory coordination refers to eliminating trajectory conflicts by optimizing the sorting trajectory. Finally, a solution without conflict obtained from the allocation result is the optimal feasible solution. The principles of the cooperative sorting of multi-arm coal and gangue sorting robots are shown in Figure 2.

The multi-arm collaborative sorting process is divided into three steps. First, the position and quality information of the gangue and the status information of the manipulator are obtained to screen the gangue that meets the distribution conditions, and the number of idle manipulators is determined. Then, the benefits of the robot arm sorting different gangue is calculated, and the distribution result in terms of priority is solved according to the task allocation algorithm. Finally, according to the trajectory constraints of the multi-arm in the execution of the task, the trajectory analysis of the allocation results is carried out according to the priority order of the allocation results to determine whether there is conflict and to determine the type of conflict, and by eliminating the conflict via task cooperation and trajectory cooperation, the feasible allocation results are calculated.

In order to facilitate processing and solving according to the working process and system working characteristics of the coal gangue sorting robot, the following descriptions are made in the research for the multi-manipulator cooperative sorting method:

1. The conveyor belt has stable working performance and moves at a constant speed without causing deviations in the gangue.
2. The coal gangue sorting robot system works reliably, regardless of the communication delay between the identification system, the control system, and the mechanical system.
3. The manipulator is isomorphic, and the properties and performance of each parameter are consistent.

## 3. Real-Time Task Allocation Method Based on Comprehensive Income

### 3.1. Mathematical Model of Task Assignment

**Figure 3:**
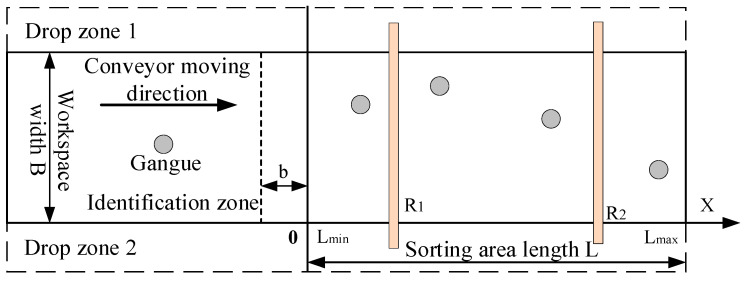
Model of coal and gangue sorting robot working area.

The working plane structure of the conveyor belt and the world coordinate system are shown in Figure 3. The identification area is the work area of the identification system, the sorting site is where the mechanical arm performs the sorting task, and the placement area is where the captured gangue is temporarily stored.

The robotic arm completes a sorting task, which involves obtaining the task, tracking, grabbing, and the placing process. The existing coal gangue sorting process calculates and grabs the gangue that has reached the sorting area. If the tracking trajectory of the robotic arm exceeds the sorting area, it will cause missed sorting. This paper proposes pre-calculating the grab points when the coal gangue is at a certain distance b from the sorting area and screening out gangue whose grab points are in the sorting area for task assignment.

The dynamic target trajectory tracking algorithm is used to calculate the gangue sorting time and grasping point of each manipulator.A gangue in which the position is beyond the sorting area is removed and defined as a missed gangue. A gangue in which the grasp point is in the sorting area is selected to participate in the task allocation and described as the gangue to be allocated. The gangue set assigned to each robot arm is defined as the target set, and the actual target to be captured by the robot arm is defined as the sorting target. The gangue that is not captured needs to be redefined as the gangue to be allocated or missed gangue according to the location information.

The mathematical description of the task assignment process is as follows:

(1) Tasker: The parameter set of the robot arm $R_{i}$ that completes the sorting work is described as follows:
  (1) $$R_{i}=(X_{i},Y_{i},Z_{i},V,A,K_{i}),i=1,2\cdots n$$
  where n is the number of arms; $X_{i}$, $Y_{i}$ and $Z_{i}$ represent the coordinate information of the end-effector of the manipulator, unit: mm; V is the running speed of the manipulator, unit: m/s; A represents the acceleration, unit: m/s^2^; and $K_{i}$ is the state quantity of the robotic arm, which indicates whether the robotic arm is in the sorting state and is defined as a 0∼1 variable, as follows:
  (2) $$K_{i}=\{\begin{array}{c} 0,\;R_{i}\;is\;idle;\\ 1,\;R_{i}\;is\;busy; \end{array}i=1,2\ldots n$$
(2) Task: Task refers to the $G_{j}$ set of gangues to be allocated, which is marked according to the sequence of gangues appearing in the direction of movement of the conveyor belt and is defined as follows:
  (3) $$G_{j}(x_{j},y_{j},z_{j},m_{j},v)\;,\;j=1,2\ldots r$$
  where $x_{j}$, $y_{j}$, and $z_{j}$ represent the coordinate information of coal gangue $G_{j}$ on the conveyor belt plane, unit: mm; $m_{j}$ is the mass of the gangue, unit: kg; v is the moving speed of the gangue, that is, the speed of the conveyor belt, unit: m/s; and r is the amount of gangues to be allocated.
(3) Decision-making: $X_{ij}$ indicates whether to assign task $G_{j}$ to tasker $R_{i}$, and it is defined as a 0∼1 variable, as follows:
  (4) $$\begin{array}{l} X_{ij}=\{\begin{array}{cc} 0, & \;G_{j}\;isn’t\;assigned\;to\;R_{i};\\ 1, & G_{j}\;is\;assigned\;to\;R_{i}; \end{array}\\ i=1,2;j=1,2\ldots r \end{array}$$
(4) Constraints: The critical value of the safety distance between the manipulators is 0.2 m, and the real-time distance between the manipulators is represented by d(d≥0), which is calculated by the difference between the abscissas of the manipulators at the same time node. The calculation equation is as follows:
  (5) $$d=X_{2}-X_{1}$$

The coordinates of the gangue to be allocated meet in real time:

(6) $$\{\begin{array}{cc} L_{\min}+b<x_{j}<L_{\max}, & j=1,2\ldots r\\ 0<y_{j}<B, & j=1,2\ldots r \end{array}$$

The coordinates of the robotic arm working globally satisfy the following in real time:

(7) $$\{\begin{array}{c} L_{\min}<X_{i}<L_{\max},\\ d\geq 0.2,\;\\ \;0<Y_{i}<B, \end{array};i=1,2$$

### 3.2. Construction of the Benefit Function of the Robotic Arm

The sorting of gangues by a mechanical arm is a continuous process. If the time taken to sort gangues with a robot arm is considered the minimum, the sorting times of the robot arm will increase in unit time, but we cannot guarantee that the total mass of the gangues sorted by the robot is large. If only the sorting of a large quality of gangue is considered, the average sorting time of the manipulator is too long, and the sorting efficiency of the robot is reduced. This paper comprehensively considers the quality of the gangue and the time required for the robotic arm to sort the gangue and selects the gangue according to characteristics such as a short sorting time and good gangue quality by the manipulator.

The benefit $C_{ij}$ of manipulator $R_{i}$ for sorting gangue $G_{j}$ takes the income per unit time as the evaluation standard, so the benefit function of mechanical arm $R_{i}$ is defined as the ratio of income $S_{ij}$ to consumption time $T_{ij}$, and the calculation equation is as follows:

(8) $$C_{ij}=\frac{S_{ij}}{T_{ij}}$$

The quality of coal gangue can be estimated by the recognition system. Define the revenue $S_{ij}$ of the robotic arm $R_{i}$ for sorting the gangue $G_{j}$ as the mass $m_{j}$ of the gangue. The equation is as follows:

(9) $$S_{ij}=m_{j}$$

The time $T_{ij}$ needed by the manipulator $R_{i}$ to sort $G_{j}$ of gangue can be calculated in advance by the dynamic target trajectory tracking. The sorting process is divided into tracking, grasping, and placing. When the manipulator reaches the grasping point within the allowable error range, it is considered to have completed the target tracking and catching. Then, when it comes to the placing point, it is deemed to have completed a sorting task.

The real-time position of gangue is calculated when the initial position of gangue is known and the conveyor belt is under constant velocity. The PID trajectory tracking algorithm calculates the position and velocity deviation between the manipulator’s end-effector and gangue in real time [12,25]. The system obtains the current position of the mechanical arm through the encoder on the mechanical arm, corrects the calculation deviation, and forms a closed-loop control. The position and speed of the end of the mechanical arm can be controlled in the x, y, and z directions to make the end quickly approach the target and to complete the grasping and placement tasks.

**Figure 4:**
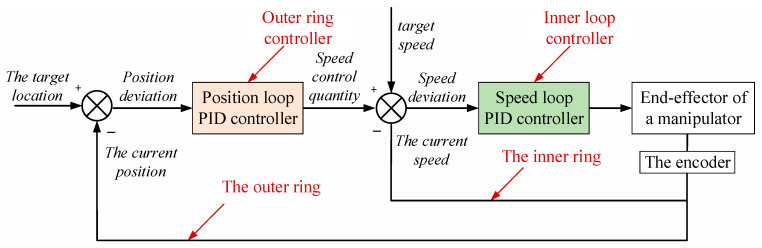
PID tracking algorithm control schematic diagram.

Dynamic target tracking adopts the outer loop position PID algorithm and the inner loop speed PID algorithm. Each comprises three parts: proportional control, integral control, and differential control. The control principle is shown in Figure 4. The input target position of the outer ring position control is the desired position; that is, the real-time position of the gangue fed back by the system at the current moment. The input of the inner loop controller is the speed control quantity obtained by the position loop PID controller and differential calculation. The speed loop PID controller adjusts the speed control quantity according to the deviation between the target speed and the actual speed monitored at present. The encoder can monitor and calculate the current position and speed of the mechanical arm, forming a feedback loop. Through the joint action of the two controllers, the end of the manipulator can approach the target smoothly and quickly to complete grasping tasks.

The positional discrete PID control equation in practical applications is as follows:

(10) $$s(k)=k_{p}\left[e(k)+\frac{T}{T_{i}}\sum_{i=0}^{k}e(i)+\frac{T_{d}}{T}(e(k)-e(k-1))\right]=k_{p}e(k)+k_{i}\sum_{i=0}^{k}e(k)+k_{d}[e\left(k\right)-e(k-1)]$$

where s(k) is the position control displacement, which controls the displacement of the three axes of X, Y, and Z; e(k) is the deviation between the expected value and the current value; T is the sampling interval; $k_{p}$ is the proportional coefficient; $T_{i}$ is the integral time constant; $T_{d}$ is the differential time constant;$k_{i}=k_{p}\times T/T_{i}$ is the integral coefficient; and $k_{d}=k_{p}\times T_{d}/T$ is the differential coefficient.

Similarly, the equation for speed control is as follows:

(11) $$v(k)=kpev(k)+k_{i}\sum_{i=0}^{k}e_{v}(k)+K_{d}[e_{v}(k)-e_{v}(k-1)]$$

**Figure 5:**
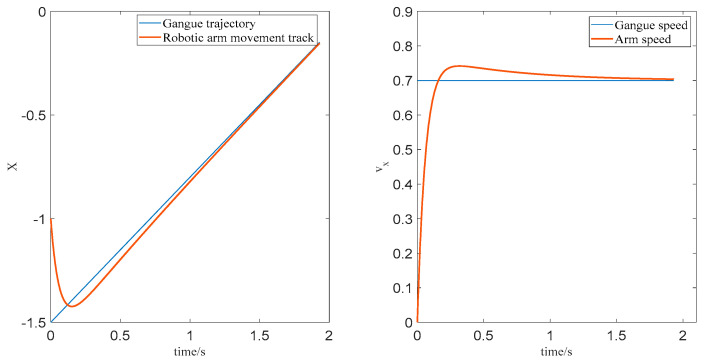
X−axis position and velocity change curve.

The trajectory tracking process of the end effector of the robotic arm in the X−axis direction is shown in Figure 5. The tracking process in the Y−axis and Z−axis directions is the same as the curve change principle of the x-axis.

The robotic arm $R_{i}$ tracks the grasping time $t_{ig}$; takes the maximum values of the X−axis motion time $t_{ix}$, Y−axis motion time $t_{iy}$, and Z−axis motion time $t_{iz}$ of the robotic arm; and defines $t_{ij}$ as the sum of the robotic arm tracking and grasping time $t_{ig}$ and placement time $t_{if}$, expressed as follows:

(12) $$t_{ig}=\max(t_{ix},t_{iy},t_{i}{}_{z})$$

(13) $$T_{ij}=t_{ij}=t_{i}{}_{g}+t_{if}$$

Combined with Equations (8)–(13), the calculation equation of benefit $C_{ij}$ of manipulator $R_{i}$ sorting gangue $G_{j}$ is as follows:

(14) $$C_{ij}=\frac{S_{ij}}{T_{ij}}=\frac{m_{j}}{t_{ij}}$$

The task assignment is to make the total benefit of the manipulator the highest. That is, the objective function is as follows:

(15) $$\max Z=\sum_{i=1}^{n}\sum_{j=1}^{r}C_{ij}X_{ij}$$

(16) $$s.t.\;\sum_{j=1}^{r}Xij\leq 1,i=1,2\cdots n;$$

(17) $$\begin{array}{l} \sum_{i=1}^{n}Xij\leq 1,j=1,2\ldots r;\\ Xij=0or1,i=1,2\cdots n,j=1,2\cdots r; \end{array}$$

where (16) means that the robotic $R_{i}$ can complete one of the r tasks at most and (17) means that the $G_{j}$ can only be completed by one of the robotic arms at most.

### 3.3. Improved Hungarian Algorithm to Solve Multi-Task Assignment

**Figure 6:**
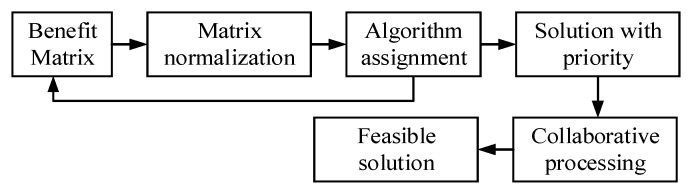
Process for improving the Hungarian algorithm.

The coal gangue sorting process requires the mechanical arm to grab only one gangue at a time. Each gangue can only be allocated to one robotic arm at most, which is an assignment problem. The Hungarian algorithm is usually used to solve this problem, while the standard algorithm is suitable for the minimum cost assignment problem when the number of tasks is the same as the number of taskers; the cost matrix is square [26,27]. In actual working conditions, the number of pieces of gangue to be sorted distributed on the conveyor belt is always greater than the number of manipulators, and the study of the multi-objective task allocation of the manipulator should obtain the maximum assignment from the perspective of income, which is a nonstandard assignment problem. In addition, after the task assignment, the assignment result should take priority so that the feasibility analysis of the assignment result can be carried out. This paper builds a benefit matrix that uses the standard Hungarian algorithm but adds virtual mechanical arms and modifies matrix elements. A set of allocation results with priority can be calculated by multiple solving. Finally, feasible solutions can be obtained through collaborative processing; that is, a round of allocation is completed. The improvement process is shown in Figure 6.

The steps to improve the Hungarian algorithm are as follows:

Step 1: Build Benefit Matrix C

Assuming that the trajectory algorithm calculates the grasping time and points, five pieces of gangue $G_{j}(j=1,2\cdots 5)$ distributed in a time sequence on the conveyor belt at a particular moment are screened out and the robotic arms $R_{i}$ are all idle (the number of arms n is 2). $C_{ij}$ represents the benefit $R_{i}$ of grasping $G_{j}$, while the benefits matrix C of the established manipulator to grab the gangue is as follows:

(18) $$C=\left[\begin{array}{ccccc} C_{11} & C_{12} & C_{13} & C_{14} & C_{15}\\ C_{21} & C_{22} & C_{23} & C_{24} & C_{25} \end{array}\right]$$

Step 2: Standardize the benefit matrix

To obtain a square matrix that conforms to the standard Hungarian algorithm and a solution with priority, we add several virtual robotic arms. The corresponding benefit is 0, so that the total number of robotic arms is equal to the number of pieces of gangue to be allocated; that is, we add several 0 rows to make it a square matrix and to find the benefits matrix to be the largest element $C_{\max}$. Thus, let

(19) $$B_{\mathrm{ij}}=C_{\max}-C_{ij}$$

(20) $$B=\left[\begin{array}{ccccc} B_{11} & B_{12} & B_{13} & B_{14} & B_{15}\\ B_{21} & B_{22} & B_{23} & B_{24} & B_{25}\\ C_{\max} & C_{\max} & C_{\max} & C_{\max} & C_{\max}\\ C_{\max} & C_{\max} & C_{\max} & C_{\max} & C_{\max}\\ C_{\max} & C_{\max} & C_{\max} & C_{\max} & C_{\max} \end{array}\right]$$

Step 3: Algorithm Solving

The specific steps to solve the optimal solution matrix are:

① Subtract the minimum element of the row from the elements of each row of the matrix B, and subtract the minimum element of the column from the elements of each column to obtain the matrix  B ′;
② Cover the row or column of 0 elements with the least horizontal and vertical lines. If the number of lines is equal to n, skip step ③, and start from ④; otherwise, execute ③;
③ Find the minimum value k among the elements that are not covered by the horizontal and vertical lines, subtract k from the uncovered elements, and add k to the elements where the lines intersect; the elements that are covered but do not intersect remain unchanged. Find matrix B″, and return to ②;
④ Try assignment: find out if there is only one 0 element row (or column); mark the 0 elements in a circle; and at the same time, cross out the 0 elements in the same column (or row) as the 0 elements until all 0 elements are circled or crossed out;
⑤ The circled 0 element is assigned 1, and the remaining elements are assigned 0, creating the optimal allocation matrix $W_{r}\left(r=1,2\cdots \right)$, where r is the number of allocations made in each allocation round.

A set of solutions can be obtained for each allocation. The earlier the gangue is allocated, the higher the priority. Repeat these three steps to obtain all solutions with their priorities. The task sequence with priority obtained by a robot arm $R_{i}$ after assignment is expressed as follows:

(21) $${\mathrm{Task}}_{R_{i}}=\left(G_{a}\to G_{b}\to G_{c}\ldots \ldots \right),a,b,c\cdots \in j$$

Step 4: Collaborative processing to determine the feasible solution

The priority of task allocation is not the execution order of manipulator tasks, but the order of multi-manipulator cooperative processing. The purpose is to find the sorting target that can guarantee the system benefit and be executed from the allocation results. The priority-based multi-manipulator collaboration process is described in detail in Section 4.

Assume that the optimal matrix $W_{1}$ obtained by solving the above five pieces of gangue once by the algorithm is as follows:

(22) $$W_{1}=\left[\begin{array}{ccccc} 0 & 1 & 0 & 0 & 0\\ 0 & 0 & 1 & 0 & 0\\ 0 & 0 & 0 & 0 & 1\\ 0 & 0 & 0 & 1 & 0\\ 1 & 0 & 0 & 0 & 0 \end{array}\right]$$

The optimal matrix’s third, fourth, and fifth rows are the assignment results of the virtual robotic arm. There is no actual robotic arm to complete, and the corresponding task will not be completed first. The first two rows of the matrix show that the result of this allocation is $G_{2}\to R_{2}$, $G_{3}\to R_{1}$. For the remaining gangue $G_{1}$, $G_{4}$, and $G_{5}$, continue to repeat steps 1 to 3, assuming that the optimal matrix $W_{2}$ for the second allocation is as follows:

(23) $$W_{2}=\left[\begin{array}{ccc} 1 & 0 & 0\\ 0 & 1 & 0\\ 0 & 0 & 1 \end{array}\right]$$

The result of this assignment is $G_{1}\to R_{1}$ and $G_{4}\to R_{2}$. If $C_{15}>C_{25}$, the assignment result of $W_{3}$ is $G_{5}\to R_{1}$. Thus far, each piece of gangue has been matched with a robotic arm.

After the above gangue is solved three times, the gangue allocated first has a higher priority, so the priority of the gangue assigned by $R_{1}$ in the allocation is $G_{3}\to G_{1}\to G_{5}$, and the priority of the gangue allocated by $R_{2}$ is $G_{4}\to G_{2}$; that is, ${\mathrm{Task}}_{R_{1}}=\left(G_{3}\to G_{1}\to G_{5}\right)$, ${\mathrm{Task}}_{R_{2}}=\left(G_{4}\to G_{2}\right)$.

## 4. The Priority-Based Multi-Arm Collaborative Process

### 4.1. Construction of Multi-Robot Collaborative Sorting Model

**Figure 7:**
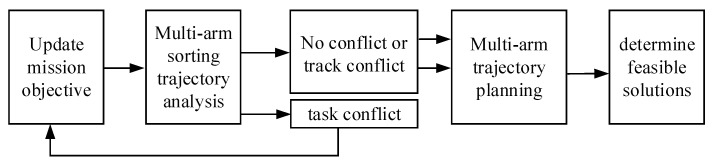
Multi-arm collaborative process.

Before the mechanical arm performs the sorting task, it must ensure that there is no collision between the robotic arms during the sorting process. The collaborative process is shown in Figure 7. Firstly, a task objective is determined according to the priority order. Then, the real-time movement trajectory of the corresponding target gangue is analyzed by multi-manipulator sorting, and whether there is conflict and the type of conflict in the sorting trajectory are judged. For the targets without or with trajectory conflict, a feasible solution can be determined by sorting trajectory planning. For task conflicts, the task needs to be updated and re-analyzed according to the task cooperation criterion until the possible solution is determined. According to the priority order of the conflict analysis, there is no need to analyze the sorting trajectory of every other gangue piece, which reduces the calculation amount in the process of determining the feasible solution; at the same time, it can ensure the system benefit and improve the sorting efficiency.

**Figure 8:**
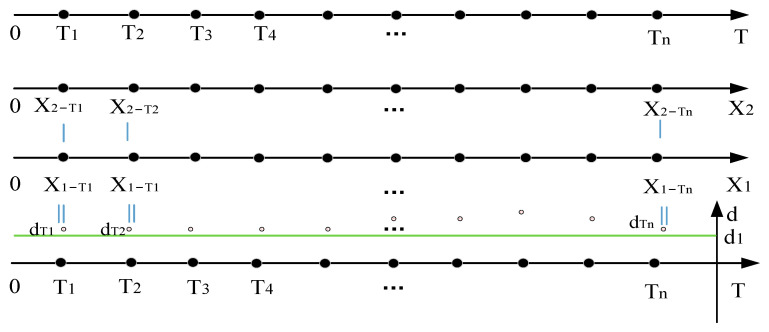
Real-time distance of the end-effector of the manipulator in the horizontal direction.

The physical structure of the manipulator adopts the cartesian coordinate formula, so it is only necessary to analyze the spatial distance of the X-axis to determine whether there is a spatial contradiction between multiple manipulators. In order to simplify the analysis process, we consider that the distance between the X-axis between the manipulators is less than the safety distance (0 ≤ *d* ≤ 2), which is the spatial overlap between the manipulators, and the overlap point is taken as the midpoint of the distance, which is marked as p. As is shown in Figure 8, the moment at which the robot arm obtains the target set after task allocation is taken as the time label, which is defined as the time starting point, 0. The longer sorting time t of the two robotic arms is the time axis T, which takes the same time interval Δt as time node $T_{i}$. $X_{1-T_{i}}$ and $X_{2-T_{i}}$ are the X-axis values of manipulator R1 and R2 at time node Ti, respectively, and $d_{1}$ (the green line in Figure 8) represents the critical values of the safety distance of the robot arm. The distance $d_{Ti}$ of the manipulator under each time node is calculated using Equation (5).

By analyzing the distance between the ends of the two manipulators at the same time node in the target tracking trajectory in the direction of movement of the conveyor belt, it is possible to determine whether there is a conflict between the manipulator’s arms during sorting, as well as the type of conflict and the time node where the trajectory conflict is located. As is shown in Figure 8, if the real-time distance $d_{Ti}$ between multiple manipulators is above $d_{Ti}$ on all real-time nodes, there is no conflict between the manipulators; if there is a node where the implementation distance $d_{Ti}$ is between $d_{1}$ and the timeline, there is a trajectory conflict, and the conflict can be eliminated by controlling the speed of the manipulator at this time node; if there is a node where the implementation distance $d_{Ti}$ is below the timeline, there is a task conflict and the conflict cannot be eliminated. The movement direction of the manipulator and the task priority of the robotic arm redefine the task.

The real-time coordinates of the robotic arms are $R_{i}(X_{i},Y_{i},Z_{i})$. According to the priority order of the assignment results, the coordinates of the task gangue grab points obtained by the robotic arms $R_{1}$ and $R_{2}$ are $G_{p}(X_{p},Y_{p},Z_{p})$ and $G_{q}(X_{q},Y_{q},Z_{q})$, respectively. When there is an uneliminable task conflict in the assignment result, the following task coordination criterion is proposed to make the assignment result a feasible solution:

(1) When the grasping trajectories of the two robotic arms are in the negative X direction at the same time, $R_{2}$ abandons the task $G_{q}$ and analyzes the next priority task. The judgment is as follows:

(24) $$\{\begin{array}{c} x_{1}-x_{p}>0,\\ x_{2}-x_{q}>0,\\ d_{Ti}<0.2, \end{array}{\;X}_{2q}=0;$$

(2) When the grasping trajectories of the two robotic arms are in the positive X direction at the same time, $R_{1}$ abandons the task $G_{3}\to R_{1}$ and analyzes the next priority task. The judgment is as follows:

(25) $$\{\begin{array}{c} x_{1}-x_{p}<0,\\ x_{2}-x_{q}<0,\\ d_{\mathrm{Ti}}<0.2, \end{array}{\;X}_{1p}=0;$$

(3) When the two robotic arms move towards each other, the robotic arm with the longest sorting time abandons the task and analyzes the next priority task. The judgment is as follows:

(26) $$\{\begin{array}{c} x_{1}-x_{p}<0,\\ x_{2}-x_{q}>0,\\ d_{Ti}\;<\;0.2,\\ t_{1p}\geq t_{2q} \end{array}X_{1p}=0;$$

or

(27) $$\{\begin{array}{c} x_{1}-x_{p}<0,\\ x_{2}-x_{q}>0,\\ d_{Ti\;}<\;0.2,\\ t_{1p}<t_{2q} \end{array}X_{2q}=0;$$

If tasks are assigned to a single robotic arm, the priorities of the tasks are sorted from high to low and analyzed and judged according to the priority and the motion trajectory of another robotic arm. When multiple manipulators are assigned tasks, according to the priority of gangue allocation to each manipulator, the grasping points of two gangue blocks and the motion trajectory of the real-time manipulator are analyzed successively until the allocation result is a feasible solution. As long as it is a feasible solution, the corresponding robotic arm can perform the sorting task without judging the subsequent ones. If there is no feasible solution, update the gangue information and enter the next round of the task allocation process.

Taking the assignment result of Section 3.3 as an example, the initial position of the end effector of the robotic arm is the position of the placement point when the last sorting task was completed (details are described in Section 4.2). In order of priority, first determine whether there is a spatial contradiction between the robotic arms in the sorting trajectories $G_{3}$ and $G_{4}$.

**Figure 9:**
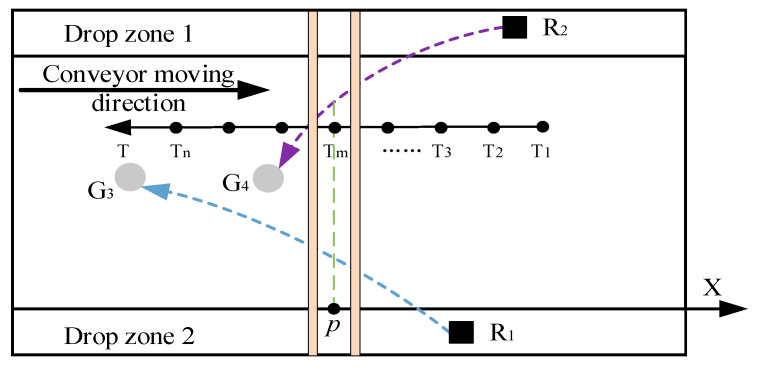
Schematic diagram of trajectory conflict of dual manipulators.

**Figure 10:**
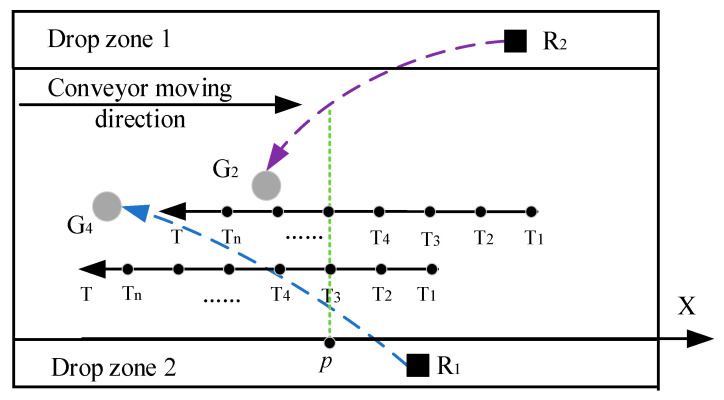
Sorting trajectory diagram 1 of the robot arm after trajectory coordination.

Assume that the actuator at the end of the mechanical arm target sorting path is as shown in Figure 9. At the time node $T_{m}$, the distance between the robotic arms is less than the safe distance, and the midpoint of the distance is p. According to the definition in this paper, the robot arm has a trajectory conflict at point p, namely the $R_{2}$ cannot pass the trajectory intersection point p. However, we can control the pace of the mechanical arm to make the mechanical arm $R_{1}$ move past trajectory intersection point p first, eliminating the trajectory conflict so that both robotic arms can complete their tasks. In this case, $G_{3}\to R_{1}$ and $G_{4}\to R_{2}$ are feasible optimal allocation results. After trajectory coordination, under the same time and space coordinates, the sorting trajectories of the two robotic arms are shown in Figure 10.

**Figure 11:**
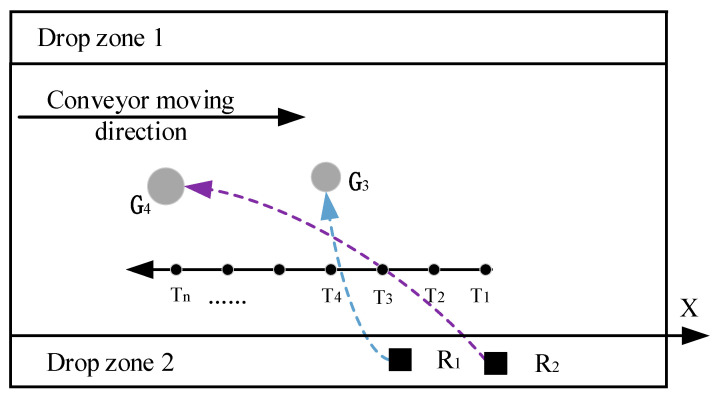
Schematic diagram of task conflict between two robotic arms.

If the trajectory of the sorting target of the robotic arm is as shown in Figure 11, it can be seen that there is a contradiction between the sorting trajectory of $R_{1}$ and the sorting trajectory of $R_{2}$, and $R_{2}$ cannot grab $G_{4}$ across the sorting space of $R_{1}$ at this time. According to task coordination criterion (1), $R_{1}$ can directly perform the sorting task, and $R_{2}$ needs to abandon $G_{4}$ to determine whether $G_{2}$ is a feasible solution.

### 4.2. Selection of Sorting Target Placement Point

**Figure 12:**
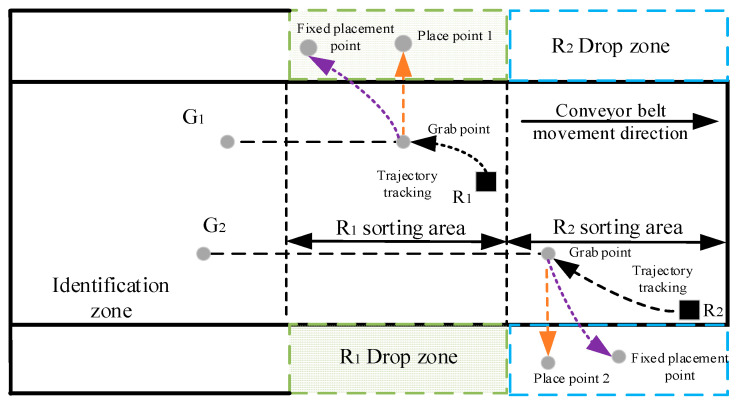
Schematic diagram of different placement points in fixed space.

After the robot arm grabs the target, it must be placed in the corresponding position to perform the next task. The existing working mode of the robot arm is generally that the position of the robot arm is fixed or has a fixed working space, and the placement point of the object is generally fixed or dynamically changing in a fixed space. The placement point diagram is shown in Figure 12.

**Figure 13:**
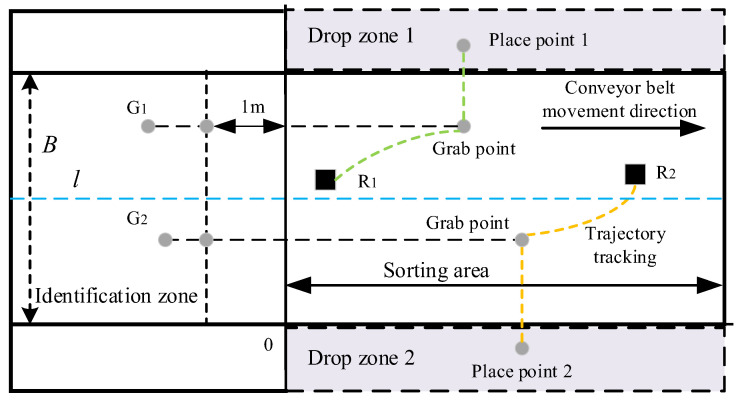
Global dynamic placement point.

In the above two working methods, the working space of the robotic arm is fixed, and the limitations are significant. The robotic arm’s sorting time and idle time are long, affecting the robot’s sorting efficiency. In order to improve the sorting efficiency of the robotic arm and to save the sorting efficiency of the mechanical arm, we put forward the working method of multi-arm global collaborative sorting. This involves selecting a placement area closer to the target gangue capture point in which to place the captured gangue. The midpoint of the placement area along the grab point perpendicular to the moving direction of the conveyor belt is the placement point; that is, the placement point is dynamically changed using a global system, as is shown in Figure 13.

**Figure 14:**
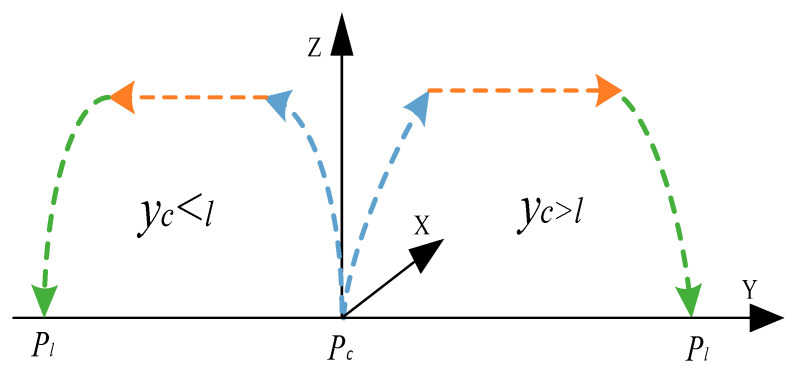
Schematic diagram of coal gangue placement process.

The placement process after the robotic arm grabs the gangue is shown in Figure 14. The end-effector moves along the Y and Z axes to reach the placement point. The coordinates of the grasping point are denoted as $P_{c}\;(X_{c},\;Y_{c},\;Z_{c})$, and the coordinates of the placement point are denoted as $P_{l}(X_{l},Y_{l},Z_{l})$. The coordinates of the placement point can be calculated from the midline l between $Y_{c}$ and the width of the workspace. The calculation equation is as follows:

(28) $$l=\frac{1}{2}\times B$$

(29) $$\{\begin{array}{c} P_{l}=(X_{c},B+\frac{1}{2}\times s,Z_{c}),y_{c}\geq l;\\ P_{l}=(X_{c},-\frac{1}{2}\times s,Z_{c}),y_{c}<\frac{1}{2}\times l; \end{array}$$

### 4.3. Evaluation Index of Multi-Manipulator Coal Gangue Sorting Result

The evaluation indicators of the task allocation results include gangue content, sorting rate, average gangue sorting time, and robot sorting capability.

According to the definition of gangue content (the percentage of gangue larger than 50 mm in the weight of raw coal), the total mass m of gangue (gangue particle size: 50–300 mm) in the raw coal samples is counted, along with the total mass M of the sample, and the gangue content is defined as follows:

(30) $$\alpha =\frac{m}{M}\times 100\%$$

The sorting results of each time are counted. At the same time, the gangue treated includes the gangue sorted and missed. The number of gangue pieces processed is $n_{sum}$, the total mass is $M_{sum}$, the number of sorted gangue pieces is $n_{deal}$, the total mass of sorted gangue is $m_{deal}$, the number of missed gangue pieces is $n_{loss}$, and the total mass of gangue that is missed is $m_{loss}$. The ratio of the total mass of the sorted gangue to the total mass of the processed gangue is the sorting rate. The calculation equation is as follows:

(31) $$\beta =\frac{m_{deal}}{m_{deal}+m_{loss}}=\frac{m_{deal}}{M_{sum}}\times 100\%$$

The average sorting time $\bar{t}(s)$ of each piece of gangue is defined as the ratio of the total running time $t_{sum}$ of the robot to the average number of gangue sorted by multiple manipulators. The calculation equation is as follows:

(32) $$\bar{t}=\frac{t_{sum}}{\frac{1}{n}\times n_{deal}}$$

Define the sorting capacity λ of the robot as the total mass $M_{sum}$ of the sorting gangue of the robot in unit time T/min. The calculation equation is as follows:

(33) $$\lambda =\frac{M_{sum}}{T}\times 100\%$$

## 5. Experiments and Results

### 5.1. Preparation of Experimental Samples

**Figure 15:**
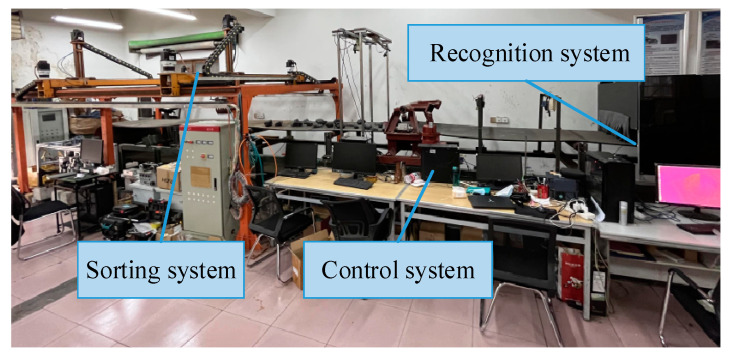
Experimental platform of multi-arm coal and gangue sorting robot.

In order to verify the efficiency and feasibility of this method, a laboratory double-arm sorting robot was used as an experimental platform and is shown in Figure 15. The robot adopts a rectangular coordinate four-degrees-of-freedom structure. The maximum speed of each axis of the robot arm is 2 m/s, and the acceleration is 4 m/s^2^. The end effector is a pneumatic manipulator.

**Figure 16:**
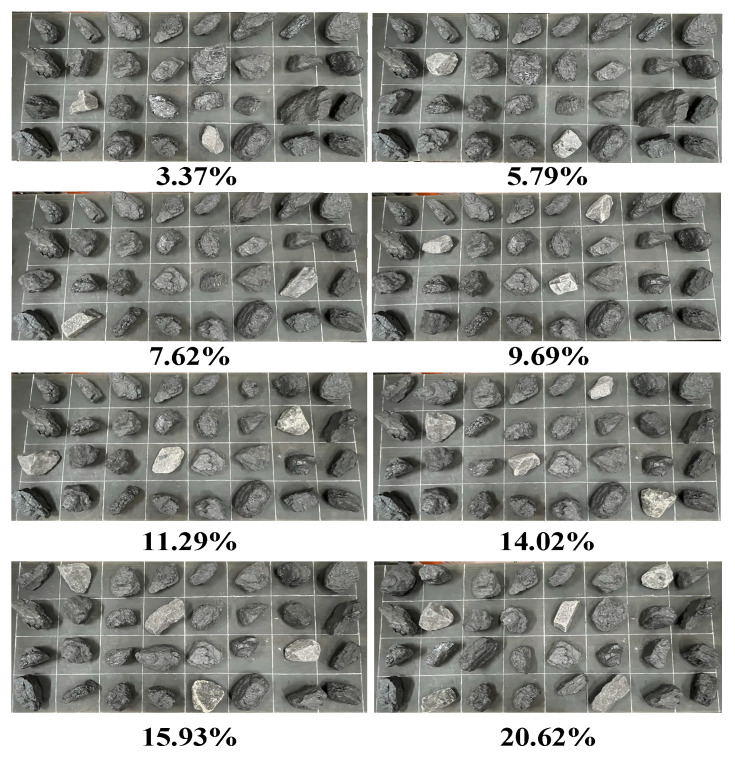
Placement diagram of samples with different gangue rates.

This paper mainly describes the problem of multi-task allocation and multi-arm cooperation. The testing samples are weighed in advance to avoid the influence of the quality error of coal gangue estimation by the recognition system on the experimental results. According to Equation (30), eight groups of experimental samples with gangue rates of 3.37%, 5.79%, 7.62%, 9.69%, 11.29%, 14.02%, 15.93%, and 20.62% with the same quality raw coal were prepared. In order to ensure the consistency of the sample data, the quality of gangue and its related parameters are randomly marked and evenly arranged in the same area of the sorting site of the experimental platform and placed on the conveyor belt in the same order. The experimental samples are shown in Figure 16.

### 5.2. Experimental Verification

On the experimental platform, the sorting conditions of the three working methods of the robotic arm were compared: ① a global work space with dynamic placement points, ② a fixed work space with dynamic placement points, and ③ a fixed work space with fixed placement point (fixed space refers to multiple robotic arms dividing the sorting area space equally. In the experimental diagram, these three working modes are simply expressed as global work space, fixed work space, and fixed placement point, respectively).

(1) Comparison of sorting effects of different sorting methods under the same gangue content and different belt speeds.

**Figure 17:**
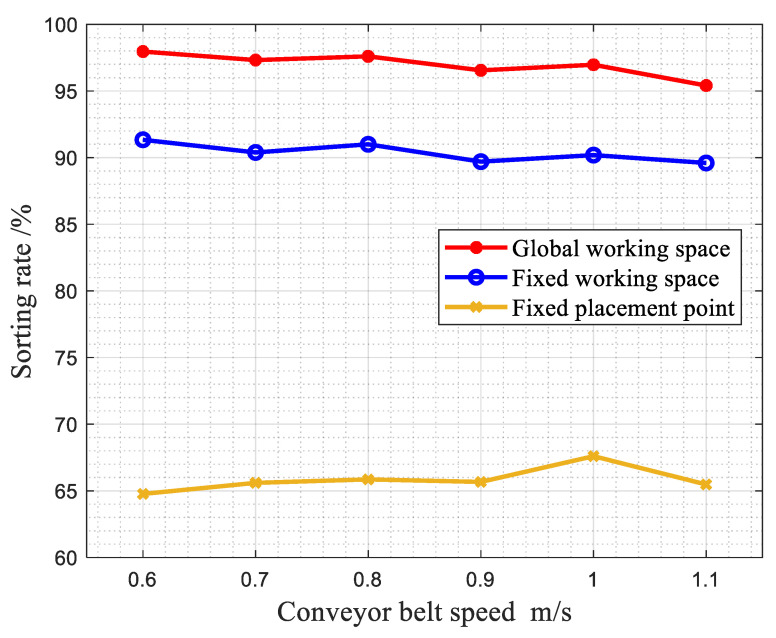
Sorting rate variation of the three sorting methods at different belt speeds.

A gangue sample with a gangue content of 5.79% was selected, and the belt speed was within a common and reasonable range (0.6~1.1 m/s). The sorting rate of the robot under the three working methods is shown in Figure 17.

It can be seen that under different belt speeds with the same set of experimental samples, the sorting rate of the robotic arm in the first working mode is always significantly higher than that in the second and third working modes. The robot is within the normal operating speed range, and the belt speed has little effect on the sorting rate of the robot.

(2) Comparison of sorting rates of different methods with different gangue contents

**Figure 18:**
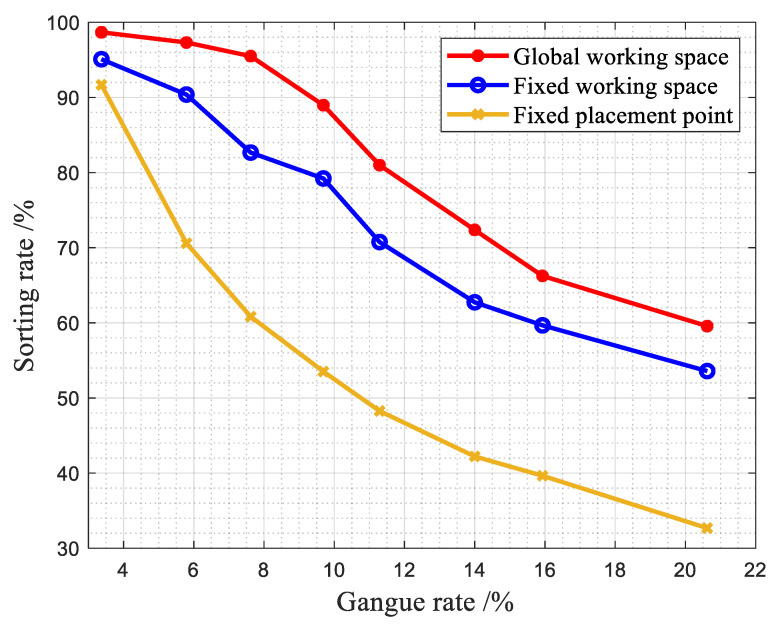
Sorting rate variation of the three sorting methods with different gangue contents.

We used the eight abovementioned groups of samples, the belt speed was 0.7 m/s, and the other experimental conditions were the same. The experimental data are shown in Figure 18.

It can be seen that the difference between the sorting rates of the sample with a gangue content of 3.37% under the three working methods of the robotic arm is small. The reason for this is that the gangue content is too low, and the sorting can be completed with a single manipulator arm. However, the robotic arm global sorting method proposed in this paper still outperforms the other two techniques. It can be seen from the rest of the samples that the sorting rate of the first working method of the robotic arm is about 10% higher than that of the second working method; the sorting rate of the second working method is higher than that of the third working method by about 20%, indicating that the proposed multi-arm cooperative sorting method based on a global working space is applicable under different gangue contents.

(3) Comparison of the average sorting time of different methods with different gangue contents.

**Figure 19:**
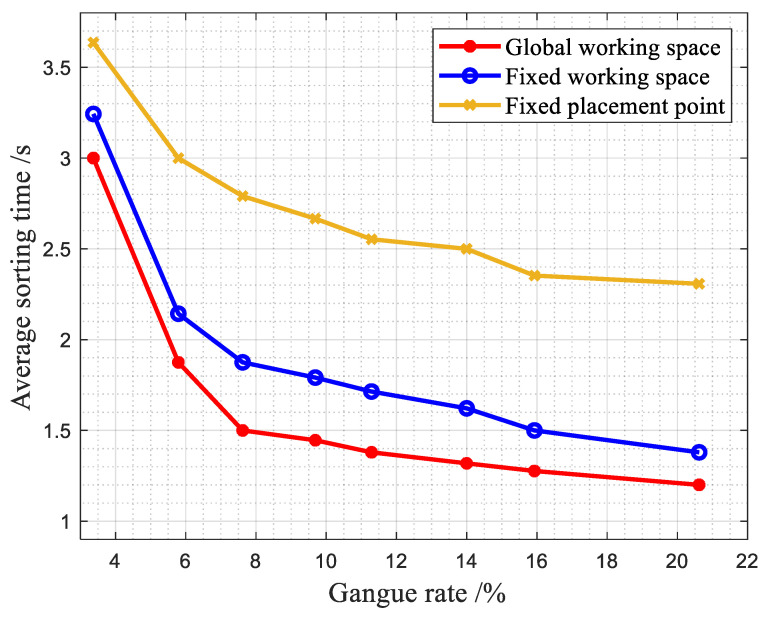
The average sorting time under the same belt speed with different gangue contents with a single arm.

In the same sample, the average time taken for a single robotic arm to sort gangue in samples with different gangue contents under the same belt speed using the three studied methods is shown in Figure 19.

According to the average sorting time of the samples, it can be seen that the average sorting time of each gangue of the first working method of the manipulator is about 0.3 s less than that of the second working method and about 1.2 s less than that of the third working method under the same belt speed.

**Figure 20:**
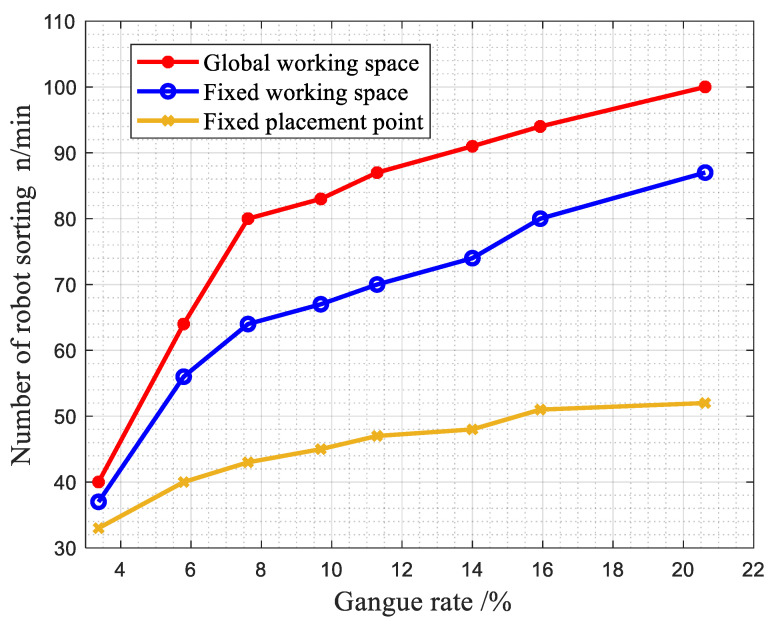
Sorting times of robots with the same belt speed and different gangue contents.

(4) The experimental data of the above samples are counted. Under the same experimental conditions, the robot’s number of pieces sorted per minute under the three working modes is shown in Figure 20.

By calculating the sorting times per minute of the robot, it can be seen that the sorting time per minute of the first working method is about 15 times/min more than the second working method and about 30 times/min more than the third working method.

(5) Comparison of robot sorting capabilities under different gangue contents and different methods.

**Figure 21:**
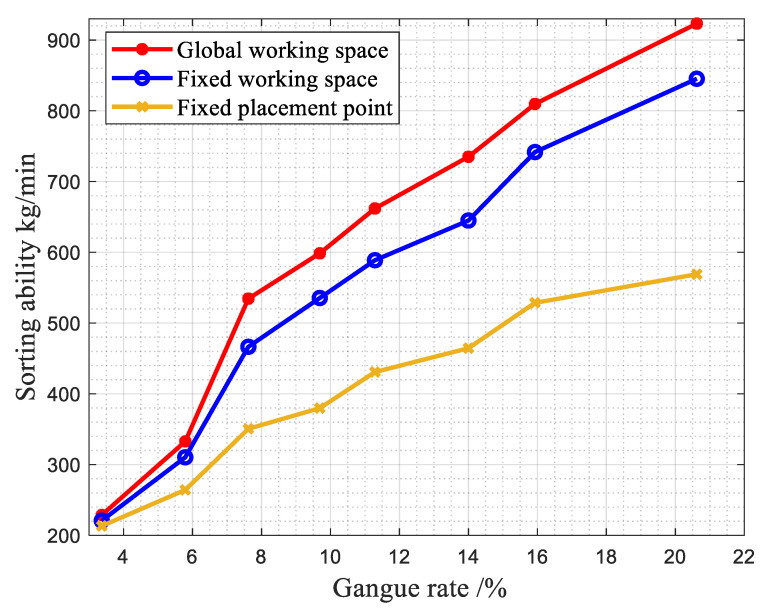
Comparison of sorting capacity at the same belt speed with different gangue contents.

The sorting ability of the robot with different gangue contents under the same belt speed with different methods is shown in Figure 21.

By calculating the robot sorting capacity of the sample, it can be seen that the sorting capability of the first working mode of the manipulator is always higher than that of the second mode and the third working mode, and it is within the limit of the capacity of the manipulator. With an increase in the gangue content, the sorting capability of the robotic arm also increases.

## 6. Conclusions

In this paper, a collaborative sorting method that uses a multi-manipulator global working space is proposed to give full play to the sorting performance of robots and to improve the sorting efficiency of robots. This paper proposes to calculate the grasping point and to screen the gangue to be allocated before the gangue enters the sorting area, and the placement point can be dynamically selected according to the grasping position, which can shorten the waiting time and sorting time of the robot arm. Based on the principle of short sorting time and great quality of gangue, the benefit function is established to ensure system benefit. In the collaborative method, an improved Hungarian algorithm is used to obtain the allocation result with priority orders. The optimal feasible solution is obtained via cooperative processing according to the order of priority. The calculation process is simple, the distribution result is optimal in real time, and the cooperation efficiency between multiple manipulators is high.

The robot system can adjust itself according to the workspace and task so that multiple robotic arms can coordinate to use one workspace, which can effectively improve the sorting efficiency of multi-manipulator coal and gangue sorting robots. Compared with a traditional manipulator that works in a fixed space with fixed placement points, the sorting rate of a manipulator working in a fixed space with dynamic placement points is about 10% higher. Compared with the working mode of each robot arm being in a fixed space with dynamic placement points, the sorting rate of having robot arms in a global working space with dynamic placement points is about 10% higher.

In the future, the idea of reinforcement learning will be applied to the collaborative sorting algorithm process so that the robot can have certain judgment, comparison, discrimination, memory, and self-adjustment abilities when interacting with the environment. This will improve the response speed and allocation efficiency of the system and help ensure a cooperative relationship between robotic arms. In addition, when the conveyor belt is running stably and the mechanical arm is always in a stable working state, we should consider adding more mechanical arms to conduct the sorting. Therefore, we will also explore the matching relationship between the number of mechanical arms and sorting efficiency in future work.

## Data Availability

Not applicable.
